# The Effects of *Berberis vulgaris* Fruit Extract on Serum Lipoproteins, apoB, apoA-I, Homocysteine, Glycemic Control and Total Antioxidant Capacity in Type 2 Diabetic Patients

**Published:** 2012

**Authors:** Farzad Shidfar, Shima Seyyed Ebrahimi, Sharieh Hosseini, Iraj Heydari, Shahrzad Shidfar, Giti Hajhassani

**Affiliations:** a *Department of Nutrition, School of Health, Tehran University of Medical Sciences, Tehran, Iran. *; b *Islamic Azad University, Sari Branch, Sari, Iran.*; c *Researches Center of Endocrinology and Metabolism, Tehran University of Medical Sciences, Tehran, Iran. *; d *University of Massachusetts, Worcester Memorial Hospital, Worcester, Massachusetts, U.S.A. *; e *Department of Pharmacology, School of Medicne, Tehran University of Medical Sciences, Tehran, Iran.*

**Keywords:** Berberis vulgaris, Diabetes, ApoB, ApoA-I, Antioxidant, Lipoproteins

## Abstract

Type 2 diabetes is a well-known endocrine and metabolic disorder which has reached epidemic proportions worldwide and represents a serious public health concern. Hyperglycemia and dyslipidemia are two major abnormalities which are major cardiovascular risk factors. Berberine is a major alkaloid in Berberis vulgaris fruit extract (BVFE) which have important role in regulation of serum glucose and fat metabolism *in-vivo* and *in-vitro* but its role in type 2 diabetes have not been extensively examined.

The aim of this study was the effect of BVFE on serum lipoproteins, apoB, apoA-I, homocysteine, glycemic control and total antioxidant capacity in type 2 diabetic patients. In a double-blind randomised clinical trial, 31 diabetic patients were randomly assigned to 3 g/d BVFE or placebo for 3 months. Serum glucose, lipoproteins, apoB, apoA-I, insulin, homocysteine and HbA1c were measured at the baseline and also at the end of the 3^rd^ month.

At the beginning and end of 1^st^, 2^nd^ and 3^rd^ months, a 24-h dietary recall questionnaire about each patients was completed. Data were analyzed by SPSS version 16.

There were significant decreases in serum TG, TC, LDL-c, apo B, glucose, and insulin and also a significant increase in TAC at the end of the study in BVFE group compared to the control group (p = 0.001, p = 0.001, p = 0.001, p = 0.001, p = 0.002, p = 0.01 and p = 0.0001 respectively).

There were significant differences in serum TG (p = 0.0001), TC (p = 0.001), LDL-c (p = 0.001), apoB (p = 0.001), glucose (p = 0.002), insulin (p = 0.01), TAC (p = 0.005), and insulin resistance (p = 0.01) between the two groups at the end of the study; but homocysteine, HbA_1c_ and HDL-c showed no significant changes between the two groups at the end of study.

The intake of 3 g/d BVFE for 3 months may have benefical effects on lipoproteins, apoproteins, glycemic control and TAC in type 2 diabetic patients.

## Introduction

It is estimated that type 2 diabetes as a serious public health concern, will affect approximatelly 366 million people by 2030 and another sobering fact is that this epidemic is being increased at a striking rate in children and adolescents (with the biggest increase in developing countries) (1-3).

Considering its devasting affects on mortality and morbidity, various combined drug therapies and lifestyle interventions have been applied to prevent or delay the progression of diabetes; however, this epidemic will probably continue to explore new therapeutic strategies (3).

All of the existing oral hypoglycemic agents have subsequent failures after a long term of administration. Thus, new oral medications are needed for long-term control of blood glucose in patients with type 2 debetes (4).

Type 2 diabetes is characterize through the increasing in insulin resistance, plasma triglyceride, apoB, homocysteine and decreasing in apoA-I and HDL-C. ApoB and apoA-I are more important predictors for preventing from cardiovascular diseases compared to the LDL-C and HDL-C, respectively (5).

Homocysteine accelerates the spontaneous athrosclerosis, reduces the concentration of circulating HDL, apoA-I and large HDL particles, inhibits the HDL function, enhances the HDL-C clearance (6), increases the associated risk of hyperlipidemia (7) and directly elicits the oxidative stress through increasing the reactive oxygen species production and diminishing the intracellular antioxidant defense.

Berberis vulgaris is a bush with yellow to brown coloured bark. The plant has obovate leaves, bearing pendulous yellow flowers in spring succeeded by oblong red coloured fruits (barberry) (8).

Berberis vulgaris fruit is safe for human consumption and is approved by FDA (9).

Berberine is the main active alkaloid with a benzyl tetra hydroxy quinoline chemical structure which can be found in all part of Berberis vulgaris especially in its fruit (barberry). The content of berberine in its fruit is 5.2%-7.7%. Barberry is not a rich source of Berberrine but is consumed widely in Iranian food plan.

Berberine is not considered toxic at doses used in clinical situations, nor has it been shown to be cytotoxic or mutagenic or having side effects.

Berberine is reported to have a comparable activity to sulphonureas or metformin. Administration of berberine was able to reduce FBG and HbA1c in adult patients with newly-diagnosed type 2 diabetes.

Berbeine has been shown to reduce the weight, enhance the insulin sensitivity, decrease the insulin resistance and decrease the blood glucose in genetic models of type 2 diabetes (10).

Berberine is demonstrated to reduce serum cholesterol, triglyceride and LDL-c in subjects with dyslipidemia and animals (11-14).

Berberine can act as a AMPK (AMP-activated protein kinase and so), increase the AMP/ATP ratio and inhibits the ATP biosynthesis in mitochondria. It can also increase the insulin sensitivity (10, 15, 16), an *α*-glucosidase inhibitor (17-19), the inhibitor of adipogenesis and it has indeed an anti-obesity activity (20-22). In addition, Berberine can increase the LDL receptor mRNA (23) and as an antioxidant, can scavenge the free radicals (25, 26). However, the majority of those researches were *in-vitro* or animal studies but the very rare clinical studies in humans were open and nonplacebo-controlled and also not consider the apoproteins, total antioxidant capacity and homocysteine.

Therefore, in the present study, we performed a randomized, double-blind, placebo-controlled trial to evaluate the effects of Berberis vulgaris fruit extract on serum lipoproteins, apoB, apoA-I, homocysteine, and glycemic control and total antioxidant capacity (TAC) in type 2 diabetic patients.

## Experimental

*Study design*

This study was a randomized, double-blind, placebo-controlled of parallel design, a 3-week run-in, and a 3-month treatment period from June 2010 to November 2010.

This study was approved by the institutional review board of Tehran University of Medical Sciences (TUMS) and the written informed consent was obtained from each patient.

The study was conducted in accordance with the principles of the declaration of Helsinki.

During a 3-week run-in period, 47 non-smoking patients who had been diagnosed with diabetes within the previous 5 years were instructed to continue their usual diet and not consuming barberry.

Forty-seven patients were randomly assigned to receive 3 g of Berberis vulgaris fruit (barberry) extract or placebo (lactose) for 3 months .

The barberry extract and placebo, were all 500 mg similar capsules, so 6 capsules were consumed each day ( two at breakfast, two at lunch, two at evening) for 3 months.

Randomization was performed centrally and was concealed and stratified in blocks of four. From 47 patients, three were lost after randomization without any visits and two dropped out with one or two visits.

A total of 44 patients, including those two who dropped out were analyzed for the intention-to-treat efficacy.

All patients were asked to maintain their usual diet and physical activity level and not to alter their life style during the intervention. Dietary intake was monitored by the same dietitian throughout the study and participants were asked to complete a 24 h diet recall questionnaire at the beginning and after 1, 2 and 3 months and a lifestyle questionnaire (*e.g*. physical activity, income) at baseline and at the end of 3 months of intervention.

The patients were followed up through phone each week; patients who had no phone were instructed to return to the clinic every other week.

*Participants*

We recruited study participants between June 2010 and November 2010 from the Endocrine Research Center (ERC) of Tehran University of Medical Sciences. Participants received no monetary incentive.

Eligibility criteria were: age of 25-70 years, diagnosed type 2 diabetes within the previous 5 years according to 1999 World Health Organisation, fasting plasma glucose ≥ 120 mg/dL or 2-h postprandial glucose ≥ 200 mg/dL (27), body mass index (BMI) < 30 Kg/m², HbA_1c_ < 9% , clinic systolic blood pressure < 180 mmHg, and diastolic blood pressure < 110 mmHg Patients were included if they were taking oral hypoglycaemic agents but not insulin.

The exclusion criteria were: a recent (within previous 3 months) or past history of symptomatic heart disase, myocardial infarction, angina pectoris or stroke, surgery, moderate or severe liver dysfunction, abnormal renal function (serum creatinine greater than 1.62 mg/dL) or thyroid disease or used nonsteroidal anti-inflammatory drugs, estrogen, progesterone or antioxidant vitamins. Patients using lipid-lowering drugs and aspirin were included but were asked not to change the dose.

*Preparation of the extract*

*Berberis vulgaris* fruit was bought from Birjand in Khorasan Province located in Iran. About 10 g of dried barberry was extracted in boiling water (100 mL, for 5 min).

The filtered aqueous extract was concentrated in a rotary vaccum evaporator and dried via exposure with hot air to yield 522 mg solid material. The stock solution of the extract (10 mg/mL) was prepared from this solid material on the day of experiment (8).

*Laboratory analyses*

Participants were required to provide venous blood samples after fasting overnight for 12-14 h on day 0 and at the end of intervention (the 3^th^ month).

All patients were required to refrain from alcohol, cigarettes and heavy physical exercise for at least 1 weak before obtaining the blood samples for biochemical measurment.

Glucose was measured immediately using an enzymatic method (CX-7 biochemical autoanalyzer; Beckman Brea, CA).

Serum insulin was measured using a double-antibody RIA (Diagnostic Systems Laboratories, Webster, TX). Serum total cholesterol and triglycerides were measured through enzymatic methods (Beckman Coulter Inc., Fullerton, CA). HDL-c and LDL-c were determined using immunoinhibition methods (HDL-c, LDL-c Direct, Wake Pure Chemical Industeris Ltd.GmbH, Neuss, Germany). HbA_1c_, homocysteine and TAC were determined using colorimetry (Pars Azmon kit, Tehran, Iran), ELISA (Randox kit, Co.Antrim, U.K.) and colorimetry (28), respectively. The within-assay and the between-assay CV(%) for these assays (n = 10) were under 1.2 and 1.4, respectively.

**Table 1:** Anthropometric data at baseline and after intervention.

**Groups**				
**Variable**	**Control (n = 21)**		***Barberry*** ** extract (n = 21)**	
	**Baseline**	**After intervention**	**Baseline**	**After intervention**
**Weight (Kg)**	75.9 ± 6.5	76.5 ± 6.7	75.2 ± 7	74.4 ± 6.2
**BMI (Kg/m²)**	27.7 ± 1	27.8 ± 1.1	27.3 ± 1	27.1 ± 1

*Statistical analysis*

Statistical analysis was performed using the PC SPSS 13.0 (SPSS Inc., Chicago, IL) and data were presented as mean ± SD.

A sample size of 21 patients in each of the two study groups, with a dropout rate up to 10%, was planned to provide a 90% power to detect a 10% or greater (with 95% confidence intervals, *α* = 0.05, *β *= 0.1) reduction in fasting serum glucose concentration (the primary end point variable) in the berberine group, compared with the placebo group after 3 months at the end of the study.

Normal distribution of the variables was checked through Kolmogorov-Smirnov test; Student’s t-test was used to test whether the differences between the mean values of the items studied in both groups were significant.

Differences in the same participants before and after 10 weeks of intervention were evaluated using paired t-test.

Diet records were analyzed by using Food Processor II software. ANOVA was employed to compare the means in different intervals of the 24 h diet recall questionnaires and also the values of within-assay and between-assay of the measurement.

For qualitative variables (*e.g*. education, occupation, income and physical activity ), a chi-square test was used.

## Results

Forty-two of forty-seven randomly assigned diabetic participatnts completed the study. Baseline characteristics of the participants confirmed that they were well matched for the inclusion criteria (age = 53.1 ± 6.3 years in barberry extract versus 52.2 ± 4.9 in control group.

The duration of diabetes were 5.5 ± 1.2 years in Berberis vulgaris fruit extract (BVFE) versus 5.4 ± 1.7 in control group.

There were no significant difference in age, duration of study, weight and BMI between the two groups (Table 1).

There was no significant difference in dietary intake of the participants in BVFE group during the study individually and also compared to the placebo group (Table 2).

The expected potential confounders to the results of the study included age and body size. None of these characteristics at baseline were significantly different between the two groups. Qualitative variables (*e.g*. education, physical activity, *etc*) measured through valid questionnire showed no significant difference between the two groups.

**Figure 1:**
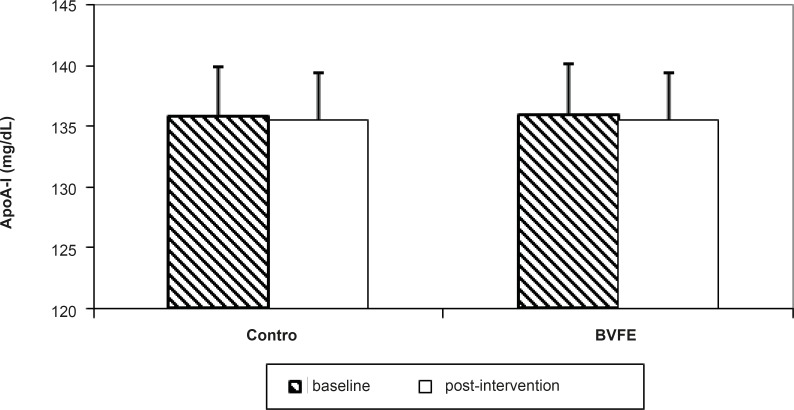
The apoA-I concentration in two groups at the baseline and post-intervention.

**Figure 2:**
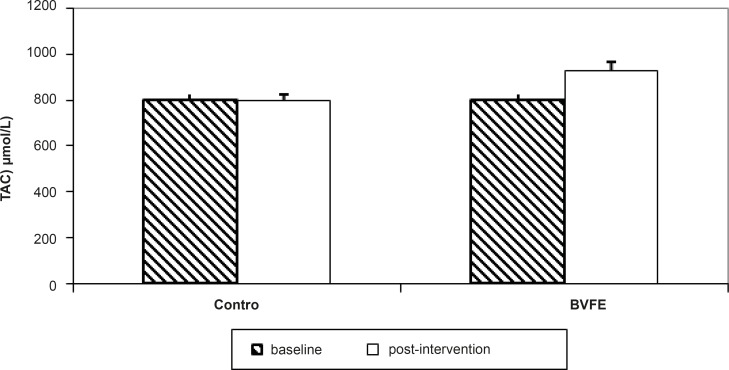
The TAC concentration in two groups at the baseline and post-intervention.

**Figure 3:**
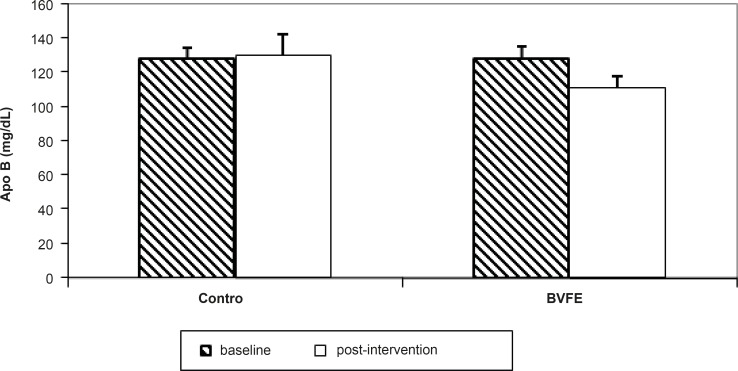
The apoB concentration in two groups at the baseline and post-intervention.

There were significant decreases in serum TG, TC, LDL-c, apo B, glucose and insulin and also significant increase in TAC at the end of the study in BVFE group compared to the control one (p = 0.001, p = 0.001, p = 0.001, p = 0.001, p = 0.002, p = 0.01 and p = 0.0001 respectively) (Table 3, Figures 1-3).

However, we found no signifcant difference in HDL-c, homocysteine and HbA1c but LDL-c/HDL-c, TG/HDL-c, apoB/apoA-I and insulin resistance had significant decreases at the end of study in BRFE group compared to the placebo group (p = 0.001, p = 0.003, p = 0.01, and p = 0.01) (Table 3).

## Discussion

The results of this randomised clinical trial demonstrated that in forty-two diabetic participants, BVFE produced significant decrease in TG, TC, LDL-c, apoB, insulin resistance, glucose, insulin and significant increase in TAC compared to the placebo group at the end of study.

To our knowledge, the present study is the first one which has compared the effect of BVFE among the diabetic patients. Significant decrease in serum TG was consistent to leng (18), Tang (19), Zhang (1) and Lee (15) studies, but all of these studies used purified berberine and were in animals but Zhang (30) and Yin (4) studies in type 2 diabetic patients, also reported significant decrease in serum TG.

However, these two studies also used purified berberine which its content was higher compared to the berberine in BVFE of our study. Wei reported the decrease of serum TG due to 0.5 g/d berberine for 3 months and indicated that the reducing effect of TG was more effective compared to reducing effect of TC which was consistent in our study. Wei indicated that the berberine effect on TG was similar to fibrate effect (31). Zhou concluded that berberine in BVFE decreases the accumulation of lipid drops in preadipocytes and inhibits the terminal differentiation of adipocyte, which may be associated with its effect on decreasing the expression of PPAR gamma 2 (proxisome proliferation activated receptor gamma 2) mRNA and protein suggesting that berberine has advantages in the decreasing of serum TG and lipid stores of diabetic patients (32).

**Table 2:** Daily dietary intake at beginning, end of 1^st^, 2^nd^ and 3^rd^ month.

**Nutrients**	**Groups**	**Basline**	**1** ^st^ ** month**	**2** ^nd^ ** month **	**3** ^rd^ ** month**
**Energy(kcal/d)**	BVFE	1435 ± 364	1470 ± 281	1450 ± 212	1433 ± 260
	Control	1428 ± 356	1463 ± 274	1442 ± 205	1426 ± 264
					
**Protein (g/d)**	BVFE	49.6 ± 6.5	47 ± 5.5	44.9 ± 5.5	45.2 ± 2.7
	Control	48.5 ± 6.1	46 ± 4.5	43.8 ± 5.1	44 ± 2.1
					
**Carbohydrate(g/d)**	BVFE	140.8 ± 18.7	138.3 ± 4.8	135.3 ± 9.1	138.4 ± 4.4
	Control	138.7 ± 18.1	137.1 ± 4.7	134.1 ± 8.1	137.3 ± 4.1
					
**Fibre (g/d) **	BVFE	3.7 ± 0.5	3.5 ± 0.4	3.6 ± 0.4	3.7 ± 0.4
	Control	3.7 ± 0.7	3.4 ± 0.5	3.5 ± 0.5	3.8 ± 0.1
					
**Total fat(g/d)**	BVFE	35.2 ± 4.0	36 ± 1.9	36.1 ± 2.1	35.1± 4
	Control	34.1 ± 3.9	35.9 ± 1.7	35.3 ± 2	35 ± 3
					
**Saturated**	BVFE	13.1 ± 1.9	13 ± 1.9	12.9 ± 1.9	12.5 ± 1.8
**Fatty acids (g/d)**	Control	13 ± 1.8	12.9 ± 1.8	12.8 ± 1.9	12.7 ± 1.9
**Monounsaturated**	BVFE	9.5 ± 0.6	9.3 ± 1	9.4 ± 1.3	9.6 ± 1.5
**Fatty acids(g/d)**	Control	9.1 ± 05	9.2 ± 1	9.3 ± 1.2	9.5 ± 1.4
**Polyunsaturated**					
**Fatty acids (g/d)**	BVFE	7.9 ± 1	8 ± 9.8	7.9 ± 0.9	7.9 ± 0.9
	Control	7.8 ± 0.9	7.9 ± 0.7	7.8 ± 0.8	7.8 ± 0.9
					
**Cholesterol (g/d)**	BVFE	39.4 ± 8.6	38.7 ± 8.3	38.9 ± 8.1	39.1 ± 8.8
	Control	39.1 ± 8.5	38.5 ± 9.3	38.8 ± 7.9	38.9 ± 8.2
					
**Vitamin c(mg/d)**	BVFE	18 ± 6.9	17.3 ± 2.5 17.1 ± 1.5	16.3 ± 2.5 16.2 ± 1.5	17.4 ± 6.6 17.3 ± 5.6
**Vitamin E(mg/d)**	Control	17.9 ± 6.2	2.6 ± 0.7	2.7 ± 0.8	2.7 ± 0.7
	BVFE	2.7 ± 0.7	2.5 ± 0.6	2.6 ± 09	2.6 ± 0.8
					
**Vitamin A (µg/d)**	BRFE	190.1 ± 60.4	227 ± 45.5	230 ± 45.5	224.8 ± 52.8
	Control	189.8 ± 60.3	220 ± 41.2	225 ± 43.1	223.1 ± 21.7

We found a significant decrease in serum TC in BVFE group compared to the control group which was consistent in the studies of Yin (4), Zhang (30) and Wei (31) in diabetic patients with 0.5 g, 1 g and 0.5 g/d, respectively, for 3 months.

It seems that the cholesterol-reducing effect of berberine was due to the up-regulation of LDL-receptor (31). Animal studies by leng (18), Lee (15) and Tang (19) were also consistent based on our study and they reported that purified berberine down-regulates the expression of genes involved in lipogenesis and up-regulates those involved in energy expenditure in adipose tissues and muscle.

Berberine treatment resulted in increased AMP-activated protein kinase (AMPK) activity in 3T3-L1 adipocytes and L6 myotubes and reduced the lipid accumulation in T3-L1 adipocytes (15).

In our study, a significant decrease in LDL-c in BVFE group compared to placebo group was consistent to Yin (4), Yin (10), Zhang (30), Tang (19) and Wei (33) which were animal studies. It seems that the benefical effect of berberine is due to the elevated low density lipoprotein receptor expression through a post-transcriptional mechanism that ehanced the stability and half-life of LDL receptor mRNA and also berberine-stabilized LDL receptor mRNA through an extracellular signal-regulated kinase-dependent pathway (33, 10).

On the other hand, berberine inhibits mitochodrial function (inhibited oxygen consumption, increase AMP/ATP ratio and AMPK activation) and this mechanism can lead to up-regulation of glucose and lipid metabolism (10). The Imanshahidi’s report was consistent to our study, however he indicated hypotensive, antiarrhythmic and lowering peripheral vascular resistance (34) which we didn’t measure it in our study.

There was no significant difference in HDL-c and apoA-I at the end of study in BVFE group compared to control group which was consistent to Yin (10) and Yin (4) but was inconsistent with Leng (18) and Tang (19) who reported the increase of HDL-c and apoA-I.

However all of studies were animal studies. It seems that higher intake of berberine in these two studies lead to significant increase in HDL-c and (18,19) apoA-I (18).

We saw significant decrease in apoB in BVFE group at the end of the study compared to placebo. Only one study which is an animal study (18), reported a similar result to our study on apoB, but no human study have assessed BVFE or purified berberine on apoB.

However, we saw no significant change in homocysteine but LDL-c/HDL-c, TG/HDL-c, TC/HDL-c and apoB/apoA-I were significantly decreased in BVFE group compared to placebo one. LDL-c/HDL-c is a well-established risk factor for cardiovascular disease (CVD) and increasing the LDL-c/HDL-c is associated with increasing the CVD but TG/HDL-c has been identified as a stronger predictor of myocardial infarction than either TC/HDL-c or LDL-C/HDL-c.

**Table 3:** Serum lipoprotein,apoproteins,glycemic control,homocysteine and total antioxidant capacity in two groups.

**Variable**	**Control** **Beseline**	**Group (n=21)** **After-intervention**	**BVFE** **Baseline**	**Group (n=21)** **After-intervention**
TG (mg/d)	201.6 ± 3.7	203.3 ± 3.7	200.8 ± 4.1	171.3 ± 3.1A,B
TC (mg/d)	203.4 ± 8.6	205.8 ± 7.19	203 ± 8.6	180.1 ± 7.5A,B
LDL-c(mg/d)	123.8 ± 6.1	125.5 ± 9.2	123.8 ± 7	106.9 ± 4.5A,B
HDL-c(mg/d)	40 ± 2.4	39.7 ± 2.6	40.6 ± 2.2	40.8 ± 2.4
LDL-c/HDL-c	3.1 ± 0.2	3.1 ± 0.2	3 ± 0.2	2.6 ± 0.2A,B
TG/HDL-c	5 ± 3.6	5.14 ± 0.3	4.9 ± 0.3	4.2 ± 0.2C,D
apoB (mg/d)	128.4 ± 6.3	130 ± 7	128.1 ± 6.8	111.2 ± 6.5A,B
apoA-I(mg/d)	135.8 ± 3.9	135.5 ± 4.4	136 ± 4.2	135.5 ± 4
Homocysteine(µmol/I)	15.2 ± 1.1	15.3 ± 3.3	15.3 ± 1.2	15.1 ± 2.3
apoB/apoA-I	0.9 ± 0.06	0.96 ± 0.05	0.94 ± 0.06	0.82 ± 0.04E,F
TAC(µmol/I)	802.1 ± 23.2	799.6 ± 23	805 ± 24.8	925.6 ± 39.2G,H
HOMA-IR	3.5 ± 0.2	3.5 ± 0.2	3.4 ± 0.2	2.3 ± 0.1E,F
HbA1c(%)	7.36 ± 1.3	7.25 ± 1.2	7.4 ± 1.5	7.2 ± 1.3
Glucose(mg/d)	140.2 ± 6.2	141 ± 7.5	140.3 ± 7.70	117 ± 5.7J,I
Insulin(mIu/mL)	10.1 ± 1.4	10.2 ± 1.3	10. ± 1.1	8.2 ± 1.5 E,F

A: p = 0.001      students      t-test      B: p = 0.001      paired      t-test

E: p = 0.01        students      t-test      F: p = 0.01         paired      t-test

G: p = 0.0001    students      t-test      H: p = 0.0001   paired      t-test

I : p = 0.002      students      t-test      J : p = 0.0002    paired      t-test

In addition, high serum TG with low serum HDL concentration (in diabetic patients) have an important role in transition from atheroma to athrothrombosis.

Small dense LDL particles are associated with an increased risk of coronary artery disease and an increase in plasma TG concentration. Both TG and HDL-c are the major determinants of LDL particle size partly for the exchange of TG from VLDL for cholesterol ester in LDL, which is mediated by cholestryl ester transfer protein (CETP). It is possible that as serum TG decrease after the BVFE intake, fewer TGs are transferred to LDL by CETP, reducing the formation of TG-enriched LDL, which minimize the opportunity for lipoprotein lipase to convert large LDL particles to small LDL ones (28).

Decreasing the TG /HDL-c and apoB/ apoA-I in our study means that the small dense LDLs are decreased significantly and coronary artery disease and myocardial infarction risk in BRFE group is much lower than control one.

Decrease of serum glucose in our study was consistent to Zhang (1) and Wei (33) in diabetic patients. Zhang announced that the hypoglycemic effect of berberine is due to inducing glycolysis and inibiting glucose oxidation in mitochondria and activating the AMP-activated protein kinase (AMPK) pathway (1).Wei indicated that the decrease of serum glucose was due to the lowering serum RBP4 levels and up-regulated the expression of tissue GLUT4 protein. RBP4 was highly related to the insulin resistance (IR) and type 2 diabetes mellitus and the serum GLUT4 levels were inversely correlated with the expression of tissue GLUT4 protein.

So, lowering the serum RBP4 levels may be an effective strategy for the prevention and treatment of type 2 diabetes mellitus (33). Leng (18), Tang (19), Lee (15), Yin (24) studies, similar to Wei (33) were consistent with our study, however all of these were animal studies but Zhang (30) and Yin (4) in diabetic patients also verified the result of our study.

We saw significant decrease of serum insulin in BVFE group compared to control ones, which was consistent to Yin (24).

He indicated that the increase of glycolysis (which is likely a consequence of glucose oxidation inhibition in mitochondria), increase of lactic acid production, activation of AMPK and increase of AMP/ATP (activation of AMPK was proposed to be responsible for the induction of glucose uptake in muscle cells) were proposed mechanism for improving the glucose metabolism.

Imanshahidi also verified the result of our study. He indicated that the Berberine effect on insulin is similar to metformin on improving insulin sensitivity (34). Yin also in type 2 diabetic patients reported that Berberine may change the body fat distribution which leads to decreasing the insulin resistance and increasing the insulin sensitivity and also to the improvement of insulin secretion, but the mentioned study had no control group.

Kong (36), Leng (18), Tang (19), Turner (37) and Zhang (1), which were animal studies, were consistent with our results. Berberine in Berberis vulgaris fruit increases the mRNA of insulin receptors and protein synthesis which is dose- and time-dependent.

Indeed, berberine may increase the insulin receptor gene expression by the way of activating its promotor which is dependent to the protein kinase–c (36). Others revealed that berberine can activate the AMP-activated protein kinase and facilitate the GLUT4 translocation, promoted glucose uptake in HepG2 cells independent of insulin action, can effectively inhibit sucrase, maltase and *α*-glucosidase to reduce glucose absorption (30).

It has also been demonstrated that inhibiting the phosphorylation of IKK*β* might be a cofactor of berberine in achieving its insulin-resistance-improving effects.

The latest study also proved that berberine could inhibit the fructose-induced insulin resistance in rats possibly through increasing the HNF-4*α* in liver. HNF-4*α* is a positive regulator of PEPCK, so an increase of HNF-4*α* would result the increased gluconeogenesis which lead to the decrease of insulin resistane (1). In spite of our results, Zhan (30) and Yin (35) reported significent decrease of HbA1c in diabetic patients, however, the dose and duration of these studies(1 and 1.5g/d purified berberine for 3 months, respectively) were higher compared to our study. HbA_1c_ in diabetic patients is higher than healthy humans that have reverse association with total antioxidant capcity (TAC).

Indeed, the increase of glucose oxidation and protein glycolysation occurs in diabetes. Finally, this glycolysation leads to the increased production of free radicals (2).

To our knowledge, there was no study to evaluate the Berberis vulgaris fruit or purified berberine on TAC, however, we saw significant increase of TAC in diabetic patients compared to the control ones.

Hyperglycemia not only generates more reactive oxygen species (ROS), such as superoxide and hydrogen peroxide, but also attenuates the antioxidative mechnisms through the glycation of scavenging enzymes including SOD and catalase. In hyperglycemia, the polyol pathway, an alternative route of glucose metabolism, will be activated. Aldose reductase, the first and rate-limiting enzyme in the polyol pathway, is activated through the hyperglycemia resulting in the overprodutction of sorbitol and fructos.

Accumulation of intracellular sorbitol can result in an increased intracellular osmotic pressure and activation of cytokines such as TNF leading to some of the pathophysiological changes associated with diabetes.

Berberine-treatment significantly inhibited the increase in aldose reductase activity and downregulated both mRNA and protein expression of aldose reductase which inhibited the polyol pathway.

However, we have not measured each of the antioxidants (SOD and catalase (which was the limitation of study)) but TAC was decreased in our study which had similar trend to Liu’s study(38).

Imanshahidi’s (34), Tomosaka’s (25) and Yin’s (10) reports were in accordance with our study but all of them were animal studies.

We can conclude that 3 g/d Berberis vulgaris fruit extract for 3 months in type 2 diabetic patients could decrease the cardiovascular risk factors, increase the TAC and improve the glycemic control.
